# Thermal immuno‐nanomedicine: A new strategy for cancer treatment

**DOI:** 10.1002/ctm2.1256

**Published:** 2023-05-23

**Authors:** Ye Hong, Di Gao, Bin Zhao, Jinlu Ma, Zhe Yang, Hui Guo

**Affiliations:** ^1^ Center of Digestive Endoscopy Shaanxi Provincial Cancer Hospital Xi'an China; ^2^ The Key Laboratory of Biomedical Information Engineering of Ministry of Education School of Life Science and Technology Xi'an Jiaotong University Xi'an China; ^3^ Department of Medical Oncology The First Affiliated Hospital of Xi'an Jiaotong University Xi'an China; ^4^ Department of Radiation Oncology The First Affiliated Hospital of Xi'an Jiaotong University Xi'an Jiaotong University Xi'an China; ^5^ Department of Epidemiology Shaanxi Provincial Cancer Hospital Xi'an China

1

Immunotherapy is an emerging treatment modality for cancer that promotes anti‐tumour immunity by mobilizing the body's immune system to suppress and kill tumour cells. Currently, various immunotherapeutic strategies have been applied in clinical practice, including immune‐checkpoint blockade, cancer vaccines, adoptive T cell transfer (ACT)‐based therapies, oncolytic viruses, cytokine‐based therapies and so on. However, poor immunogenicity of tumours (i.e., “cold” tumours) and complex immune‐suppressive tumour microenvironment have limited the clinical effectiveness of antitumor immunotherapy. Thermal therapy, a widely used cancer treatment, primarily involves raising the temperature of the entire body or tumour tissue to treat malignant tumours through thermal effects and their secondary effects. Studies have shown that thermal therapy can not only cause direct thermal damage to tumour tissues, but also induce immunogenic cell death (ICD) of tumour cells, activate the immune response of the body, and enhance the infiltration and accumulation of immune cells or drugs in tumour tissues by expanding the intratumoral blood vessels, thereby assisting in improving the effectiveness of immunotherapy. Therefore, combined thermal therapy and immunotherapy also named “thermal immunotherapy” is a synergistic therapeutic strategy for cancer treatment with enormous clinical potential. Currently, several relevant clinical studies are ongoing and have achieved promising results. However, the non‐specific accumulation of immunotherapeutic and/or thermotherapeutic agents not only influences their anti‐tumour activities but also can result in adverse events. Therefore, the key to achieving effective combination therapy is to efficiently deliver the therapeutic agents to tumours.

With the development of nanotechnology, nanomedicines have brought new hope for cancer therapy. It can not only improve the solubility and pharmacokinetic properties of hydrophobic drugs but also use tumour cell‐specific characteristics to achieve targeted drug delivery and controlled release, thereby reducing the toxicity and side effects of drugs while improving their bioavailability and efficacy. At least 20 nanomedicines are currently in clinical use as cancer therapies globally, and more than 566 clinical trials involving anti‐cancer nanomedicines were conducted between 2016 and 2020.^1^ Some of these agents had improved efficacy when combined with immunotherapy.^2^ Thus, the application of thermal immuno‐nanomedicines can improve the efficacy of cancer treatment.

Herein, recent development strategies for nanomedicines used in various thermal immunotherapies are comprehensively summarized by researchers at the School of Life Science and Technology, Xi'an Jiaotong University.^3^ For example, nanomedicine based on thermal immunotherapy as in situ cancer vaccines can overcome the disadvantage of existing cancer vaccines in which immune responses are mounted against only a limited number of antigens. In addition, these vaccines utilize thermal therapy to trigger tumour ICD and release tumour‐specific antigens, thereby effectively enhancing the anti‐tumour immune response.^4^ In another example, immune‐checkpoint inhibitors (ICIs) are loaded into nanocarriers, which effectively increase drug aggregation at tumour sites and decrease immune‐related toxic side effects. Such nanomedicines in combined therapy act synergistically with ICIs, thus further enhancing anticancer effects while achieving antigen release and promoting T‐cell recruitment to tumour tissues through thermal therapy.^5^ Moreover, gene editing (e.g. CRISPR‐Cas9) and gene silencing (e.g. small‐interfering RNAs) technologies targeting immune checkpoints also function better with the aid of nanomedicines, and the therapeutic application of this technology has received substantial research interest. In ACT‐based therapies (such as chimeric antigen receptor T cell immunotherapy), thermal therapy disrupts the extracellular matrix and other tumour microenvironment components, thereby promoting tumour infiltration of adaptive immune cells.^6^ Moreover, hyperthermia‐induced ICD stimulates the generation of pro‐inflammatory cytokines and chemokines, thereby enhancing the recruitment of adoptive immune cells and natural immune cells in the target tissue. As a nanomedicine constructed by covalent modification of tumour necrosis factor on the basis of PEGylated gold nanoparticles for combined thermotherapy‐cytokine therapy, CYT‐6091 improves the safety, bioavailability, and therapeutic efficacy of cytokines, and remodels the tumour microenvironment by releasing cytokines.^7^

Despite the potential of nanomedicine‐mediated thermal immunotherapy to improve the efficacy of antitumor therapy, several challenges should be overcome before these strategies can be applied in clinical practice. First, the “heat sink effect” has been demonstrated to slow the increase in local tumour temperature during hyperthermia (particularly radiofrequency ablation), thereby influencing the effects of thermal therapy.^8^ Furthermore, because the interstitial hyperthermia equipment commonly used in clinical practice cannot achieve uniform heating of tumour tissue, thermal damage to surrounding normal tissues is unavoidable. Therefore, more advanced hyperthermia equipment and probes must urgently be developed to optimize the effects of thermal therapy. Additionally, to decrease hyperthermia‐induced damage to functional immune cells, the temperature of the tumour during thermal immunotherapy should not excessively high. However, due to the influence of light attenuation of biological tissue and electromagnetic interference, existing temperature monitoring technologies still exit the limitation to precisely determine the tumour temperature. Therefore, improving the temperature measurement methods suitable for tumour temperature monitoring will be necessary to improve the effects of thermal immunotherapy. Notably, the use of artificial intelligence for clinical big data analysis related to thermal immunotherapy would enable more effective evidence‐based development of individualized treatments.

The development and clinical translation of nanomedicines suitable for thermal immunotherapy are also arduous tasks. First, because the effectiveness of nanomedicines in achieving tumour enrichment through passive targeting (enhanced permeability and retention effect) and active targeting ligand modification has been questioned, more innovative strategies are required to promote the enrichment of nanomedicines in tumour tissues, such as through physical means (e.g. sub‐hyperthermia, radiotherapy, or ultrasound) to promote the permeability of the vascular wall or to generate NO, which in turn stimulates the normalization of tumour blood vessels.^9^ Second, nanomedicines are usually constructed through multi‐step or complex techniques, thus resulting in poor reproducibility of preparations and extreme difficulty in scaling up. The development of carrier materials with good biosafety will also be essential to improve the efficacy of combined therapy and decrease potential toxic adverse effects. Furthermore, ideal nanomedicines for thermal immunotherapy should be selected by evaluation of the efficacy and safety of nanomedicines in advanced animal models that faithfully recapitulate the mechanisms of human diseases.

Overall, only through the collaboration of multidisciplinary expert teams can efficient thermal‐immuno nanomedicines be developed, ultimately making them a safe and effective anti‐tumour sharp weapon in clinical practice (See Figure 1).

**FIGURE 1:**
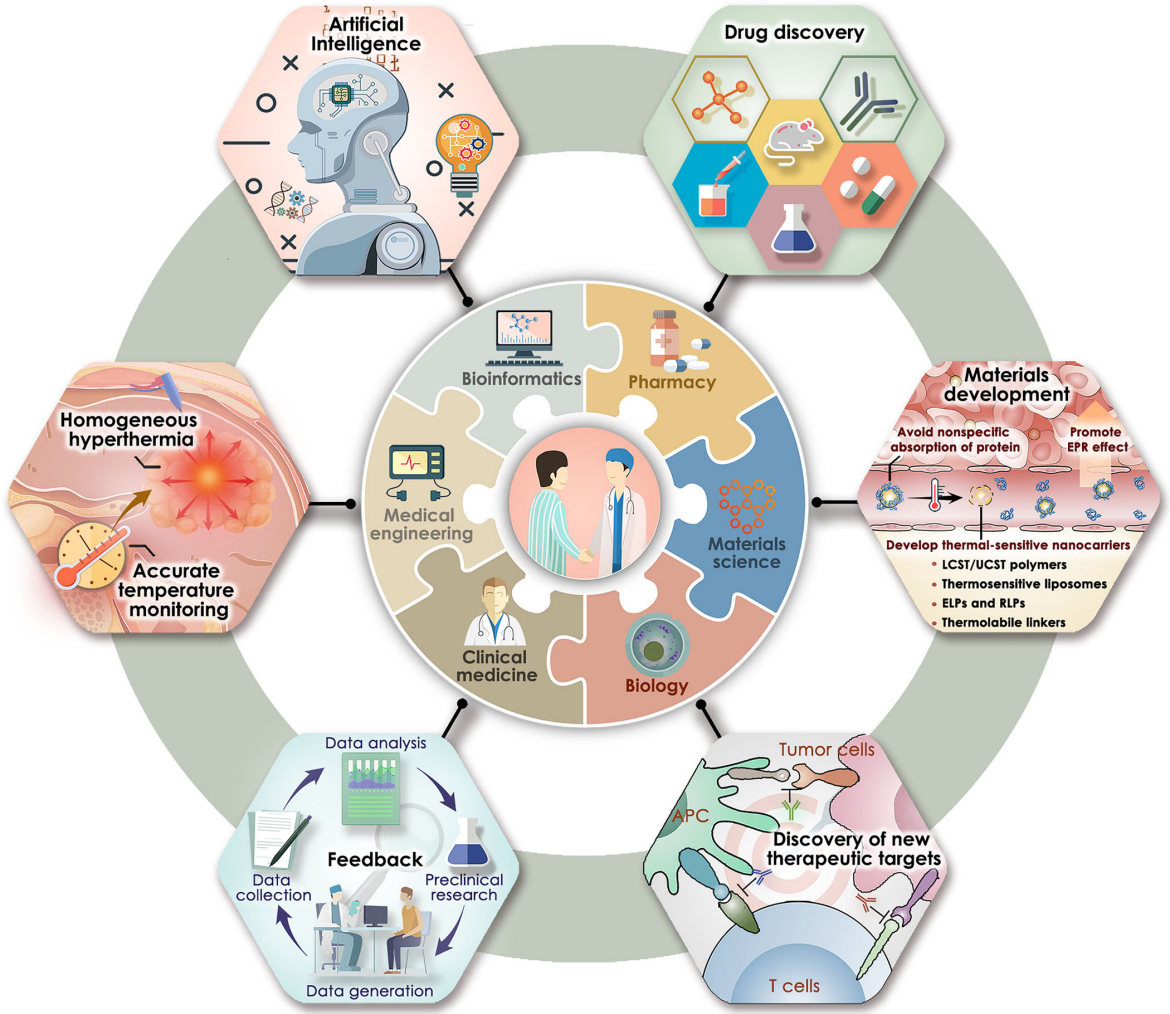
Future advancement of thermal immuno‐nanomedicines driven by multidisciplinary expert teams.

## CONFLICT OF INTEREST STATEMENT

The authors declare no conflict of interest.
